# Dual roles of BK Polyomavirus in promoting urothelial carcinoma progression via regulating CLDN1

**DOI:** 10.1186/s40364-024-00564-2

**Published:** 2024-01-20

**Authors:** Cuidi Xu, Siyue Chen, Juntao Chen, Jina Wang, Xinhao Niu, Ruiming Rong, Tongyu Zhu, Yigang Zeng

**Affiliations:** 1 Department of Urology, Zhongshan Hospital, Fudan University, Shanghai, China; 2 Shanghai Key Laboratory of Organ Transplantation, 180 Fenglin Road, Shanghai, 200032 China; 3 Department of Urology, Shanghai Public Health Clinical Center, Fudan University, 2901 Caolang Road, Shanghai, 201508 China; 4 Department of Transfusion, Zhongshan Hospital, Fudan University, Shanghai, 200032 China; 5 Department of Urology, Shanghai General Hospital, Shanghai Jiao Tong University School of Medicine, Shanghai, 200080 China

## Abstract

Uncontrolled productive infection of BK polyomaviruses (BKV) in immunocompromised patients was reported to result in serious diseases, especially renourinary malignancies. However, the mechanism of BKV as a role of human carcinogen is still unknown. In this study, we showed that there is a significant association between BKV infection and metastasis of urothelial carcinoma (UCA). BKV-infected tumor tissues exhibit invasive histologic phenomena with vascular invasion and myometrial invasion. Then we identified that BKV promotes UCA invasion in a mode of dual regulation of tumor cells (TCs) invasion and endothelial cells (ECs) adhesion by encoding miRNAs. In cancer cells, BKV-B1-miR-5p promotes cell motility and invasiveness by directly targeting CLDN1. Moreover, exosomal-BKV-B1-miR-3p derived from BK-infected BC cells would be transferred to ECs and increase its adhesion to tumor cells by switching on the CLDN1 enhancer, which subsequently destroyed endothelial monolayers and increased permeability. In a human urothelial cancer metastasis mouse model, BK-inoculated cells exhibited higher incidence of vascular leakage and liver colonization. However, the vascular leakage and liver metastasis could be reduced when knocking down miRNAs in BK-inoculated cells. Our research delineates the bifunctional impact of BKV-encoded microRNAs on the expression of CLDN1 within both TCs and ECs, which orchestrates the establishment of a pre-metastatic niche in UCA.

**Supplementary Information:**

The online version contains supplementary material available at 10.1186/s40364-024-00564-2.

To the Editor,

BK polyomaviruses (BKV) establish an asymptomatic persistent infection in the urinary system among healthy people, which is always benign but reactivates commonly in immunosuppressed individuals [1–3]. Activated BKV has been ruled as a potential oncogenic factor, particularly in urinary tract malignancies because the urothelial surface of bladder and renal pelvis constitute the primary loci for BKV’s productive replication [4]. Our previous work found that BKV promotes tumor cells motility and invasion by mediating CLDN1, which facilitates tumor aggressiveness [5]. The late region of BKV encodes a precursor miRNA transcript which produce two mature miRNAs: BKV-miR-B1-5p (BKV-miR-5p) and BKV-miR-B1-3p (BKV-miR-3p) [6]. Li et al. observed high levels of BKV-miR-5p in blood was significantly associated with high levels of BKV DNA [7]. The expression of LTAg is a crucial sign of BKV infection. LTAg-positive urothelial carcinomas showed an invasive histologic phenomena with vascular invasion (Fig. S1a). Based on these findings, we hypothesized that BKV encoded miRNAs promote tumor invasion in a dual role on TC and EC.

To test this hypothesis, HTB-9 and TCC-SUP cells were inoculated with or without BKV (MOI = 1) (Fig. S1b). In both cell lines, RT-qPCR and WB analysis showed that BKV inoculation significantly upregulated BKV-miR-5p and BKV-miR-3p and downregulated CLDN1 (Fig. S1c-S1f). A decreased invasion activity was assessed when BKV-miR-5p was downregulated (Fig. 1a, S2a). Therefore, we employed HTB-9 for further study, CLDN1 were increased when BKV-miR-5p were suppressed in BKV-inoculated cells (Fig. 1b). miRNAs were traditionally thought to inhibit gene expression by targeting 3’UTR of mRNA in cytoplasm [8]. We constructed the wild-type (WT) and mutant (MUT) 3’UTR plasmids of CLDN1 which contains the predicted 5p miRNA-binding site (Fig. S1g). Results showed that BKV-miR-5p notably inhibited luciferase reporter activity of WT 3’UTR (Fig. 1c). Additionally, blocking CLDN1 (Fig. S1h) enhanced the migration and invasion ability of tumor cells (Fig. 1d). Therefore, in TCs, BKV encoded BKV-miR-5p promotes TC invasion by directly targeting CLDN1.

**Fig. 1:**
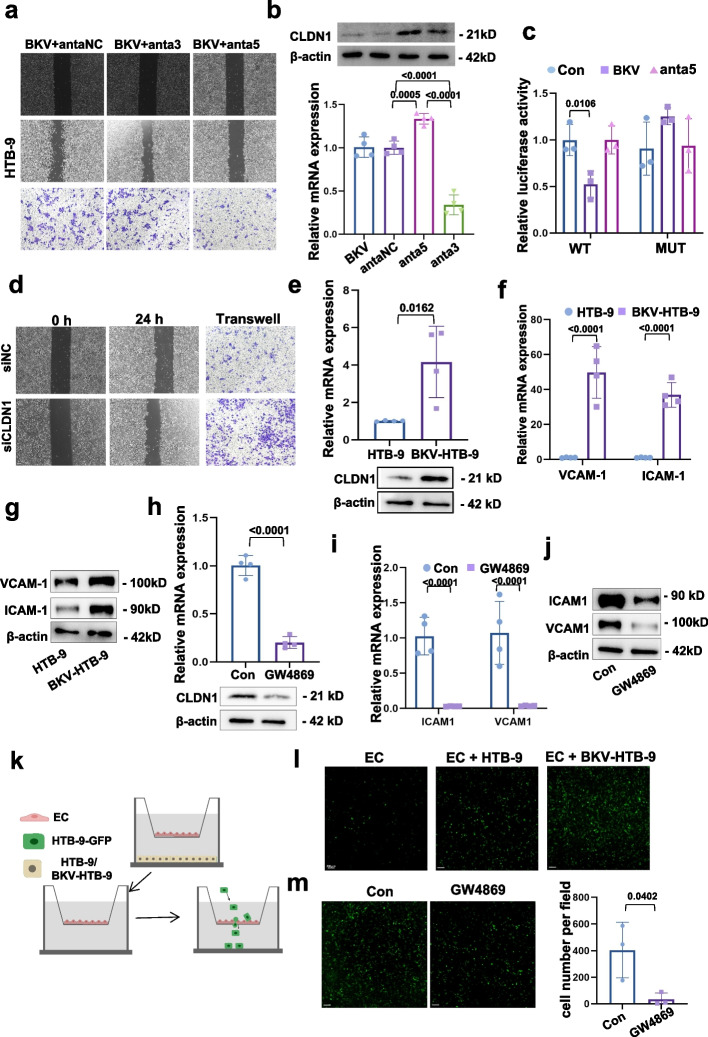
BKV encoded miRNAs promote tumor invasion in a dual role on TC and EC. **a** Migration and invasion assays of BKV-infected HTB-9 cells (BKV) after reducing BKV-miR-3p and BKV-miR-5p. **b** RT-qPCR and WB analysis shows the mRNA and protein levels of CLDN1 increases after transfecting with anta5. **c** Dual Luciferase assay shows BKV-miR-5p directly binds to 3’UTR region of CLDN1. **d** Migration and invasion assays of HTB-9 cells after downregulating CLDN1. **e** CLDN1 is increased in ECs cocultured with BKV-HTB-9. **f**-**g** ICAM1 and VCAM1 are increased in ECs cocultured with BKV-HTB-9. **h** CLDN1 expression is reversed in ECs after BKV-HTB-9 pretreated with GW4869. **i**-**j** ICAM1 and VCAM1 are reversed in ECs after BKV-HTB-9 pretreated with GW4869. **k** Experimental scheme of transendothelial invasion assay. **l** HTB-9-GFP transendothelial invasion in ECs cocultured with BKV-HTB-9. **m** HTB-9-GFP transendothelial invasion in ECs after BKV-HTB-9 pretreated with GW4869

In ECs, we purified the exosomes from conditional media (CM) of BKV-inoculated (BKV-exo) and non-inoculated HTB-9 cells (Exo) (Fig. S2b). We observed significant uptake of BKV-exo by ECs (Fig. S2c). BKV-miR-3p increased in HUVECs cocultured with BKV-inoculated HTB-9 cells (BKV-HTB-9) (Fig. S2d), but decreased when BKV-inoculated HTB-9 cells were pretreated with GW4869 (Fig. S2e). Expressions of CLDN1, ICAM1 and VCAM1 were enhanced in ECs cocultured with BKV-inoculated HTB-9 cells (Fig. 1e-g), but reversed when BKV-HTB-9 cell pretreated with GW4869 (Fig. 1h-j) or 3p miRNA antagomir (anta3) (Fig. S2f-S2h), suggesting that BKV-exo internalized by ECs affect EC adhesion to TC. Moreover, transendothelial invasion assay (Fig. 1k) showed that BKV-HTB-9 coculture promoted the invasion of HTB-9-GFP cells through ECs monolayers (Fig. 1l), but the number of invasive HTB-9-GFP decreased when the cocultured BKV-HTB-9 pretreated with GW4896 (Fig. 1m), anta3 (Fig. S2i) or siCLDN1 (Fig. S2j). Therefore, exosomal-3p secreted by BKV-inoculated cells promotes TCs adhesion to ECs, and induces monolayer leakiness.

By using si-importin8 to silence the expression of importin8, which is regarded as the transport of mature miRNAs into nucleus, we found the level of BKV-miR-3p in nucleus was significantly reduced and CLDN1 mRNA and protein were no longer promoted after reducing BKV-miR-3p in nucleus (Fig. S3a-S3c). Yu et al. declared that there also exist NamiRNAs in the nucleus co-activating target gene with enhancer [9]. Since multiple binding sites of BKV-miR-3p and CLDN1 DNA sequence were obtained from RNAhybrid algorithm (Fig. S3d), suggesting that BKV-miR-3p might function as NamiRNA. Luciferase report showed an increase in Lenti-3p and pGL3-CLDN1 group (Fig. S3e, 2a). However, the luciferase activity was decreased when we mutated the seed sequence of BKV-miR-3p (mut-miR-3p) and its corresponding complementary sites in the CLDN1 enhancer region (mut-pGL3-CLDN1) (Fig. 2a). The enrichment of H3K27ac became higher when ECs were incubated with BKV-HTB-9 derived exosomes, but reversed after pre-transfection with anta3 (Fig. 2b). Furthermore, H3K27ac CUT&TAG exhibited BKV-exo group a higher enrichment in the CLDN1 enhancer region compared with exo group (Fig. 2c). Finally, by employing CRISPR/Cas9 system, we deleted the enhancer region of BKV-miR-3p in HEK293T cells and found CLDN1 was not activated after removing the enhancer region (Fig. 2d).

**Fig. 2:**
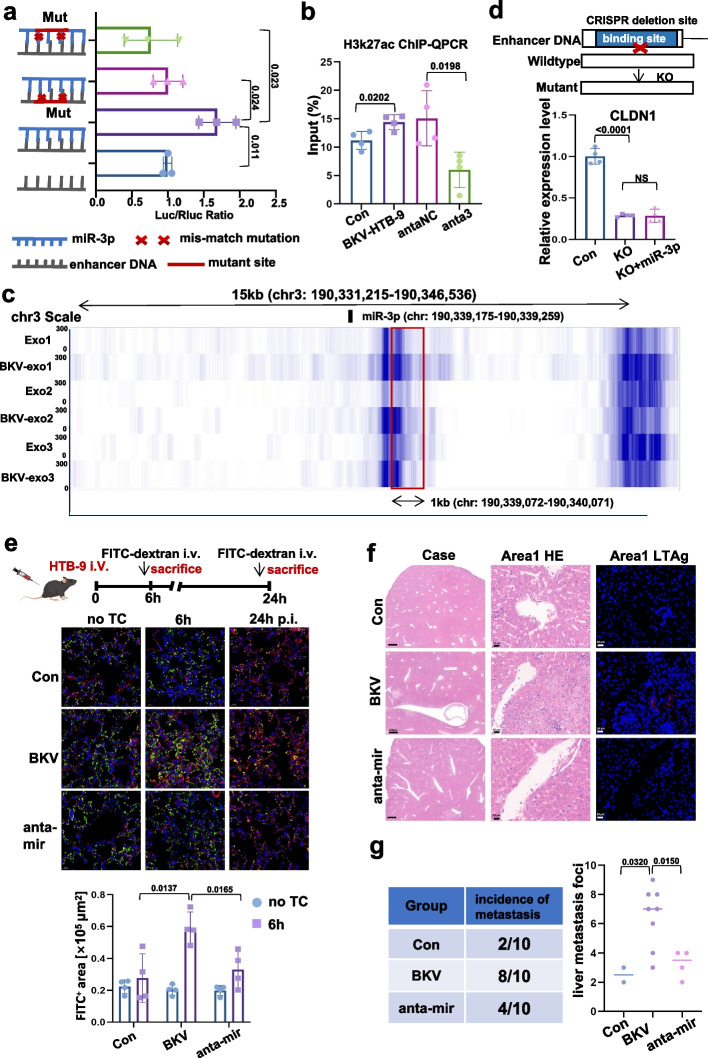
Exosomal-miR-3p promotes CLDN1 depended on enhancer elements. **a** The luciferase activity is increased when HEK293T cells co-transfecting with BKV-miR-3p and pGL3-CLDN1. The luciferase activity decreased once BKV-miR-3p and CLDN1 is mismatched. **b** An enrichment of H3K27ac in enhancer region containing BKV-miR-3p binding site exhibited by ChIP-qPCR. **c** An enrichment of H3K27ac in enhancer region containing BKV-miR-3p binding site exhibited by CUT&TAG sequencing. **d** Knockout of binding site by CRISPR/Cas9 fails to promote CLDN1 expression. **e** Representative images of lung sections stained for indicated markers. FITC + positive area is quantified as a measure of vascular permeability. BKV-inoculated cells enhance vascular leakage, which was counteracted if knockdown BKV-miR-3p and BKV-miR-5p. **f** H&E staining of Liver sections. Area 1 in BKV group with positive expression of LTAg indicates that tumor cells invade blood vessel. **g** Statistics of liver metastasis in Con, BKV and anta-mir groups

Finally, we validate the in vitro result in vivo. BKV-inoculated tumor had more Ki67 positive cell compared with Con group. H&E staining showed BKV tumors displayed spike-like structures that invaded the surrounding tissues, while the Con group showed sharp edges (Fig. S3f, S3g). RT-PCR and WB analysis both showed that tumors formed by BKV-inoculated cells exhibited lower expression of CLDN1 Fig. S3h). Then, BKV-inoculated HTB-9 cells were injected into C57BL/6 mice. As determined by FITC-dextran egress in lung, mice injected with BKV-inoculated HTB-9 cells exhibited higher vascular leakage. Conversely, silencing BKV-miR-3p and BKV-miR-5p could reverse the effect of BKV in vascular leakage (Fig. 2e). Secondly, we injected TCs into BALB/c nude mice via tail veins. BKV group exhibited positive LTAg staining and vascular invasion (Fig. 2f). The liver metastasis incidence of BKV-inoculated cells was 80% versus 20% of non-inoculated cells. Meanwhile, when knocking down the BKV-miR-3p and BKV-miR-5p, the incidence of liver metastasis decreased to 40% (Fig. 2g). These findings supported that BKV-encoded miRNAs exhibit dual regulation on cancer invasion.

### Supplementary Information

**Additional file 1.**

## Data Availability

The datasets used and /or analyzed during the current study are available from the corresponding author on reasonable request.

## References

[1] Peretti A, Geoghegan EM, Pastrana DV, Smola S, Feld P, Sauter M (2018). Characterization of bk polyomaviruses from kidney transplant recipients suggests a role for apobec3 in driving in-host virus evolution. Cell Host Microbe.

[2] Gras J, Le Flecher A, Dupont A, Verine J, Amara A, Delaugerre C (2023). Characteristics, risk factors and outcome of bkv nephropathy in kidney transplant recipients: a case-control study. Bmc Infect Dis.

[3] Agosto-Arroyo E, Coshatt GM, Winokur TS, Harada S, Park SL (2017). Alchemy: a web 2.0 real-time quality assurance platform for human immunodeficiency virus, hepatitis c virus, and bk virus quantitation assays. J Pathol Inform.

[4] Tsai HL, Chang JW, Wu TH, King KL, Yang LY, Chan YJ (2014). Outcomes of kidney transplant tourism and risk factors for de novo urothelial carcinoma. Transplantation.

[5] Zeng Y, Sun J, Bao J, Zhu T (2020). Bk polyomavirus infection promotes growth and aggressiveness in bladder cancer. Virol J.

[6] Demey B, Descamps V, Presne C, Helle F, Francois C, Duverlie G (2021). Bk polyomavirus micro-rnas: time course and clinical relevance in kidney transplant recipients. Viruses.

[7] Li JY, Mcnicholas K, Yong TY, Rao N, Coates PT, Higgins GD (2014). Bk virus encoded micrornas are present in blood of renal transplant recipients with bk viral nephropathy. Am J Transplant.

[8] Zhuang J, Shen L, Li M, Sun J, Hao J, Li J (2023). Cancer-associated fibroblast-derived mir-146a-5p generates a niche that promotes bladder cancer stemness and chemoresistance. Cancer Res.

[9] Liang Y, Lu Q, Li W, Zhang D, Zhang F, Zou Q (2021). Reactivation of tumour suppressor in breast cancer by enhancer switching through namirna network. Nucleic Acids Res.

