# Supplementary material for: Dual roles of BK Polyomavirus in promoting urothelial carcinoma progression via regulating CLDN1
Source: Biomark Res. 2024 Jan 20;12:9. doi: 10.1186/s40364-024-00564-2 (PMC10800034; doi:10.1186/s40364-024-00564-2)
Supplement: Supplementary file 1 — Additional file 1. [file 40364_2024_564_MOESM1_ESM.pdf]

Supplementary Information

BKV promotes urothelial carcinoma invasion and metastasis by encoding miRNAs

Cuidi Xu[1#], Siyue Chen[1#], Juntao Chen[1#], Jina Wang[1], Xinhao Niu[1], Ruiming Rong[1,3*], Tongyu Zhu[1*], Yigang Zeng[2*]

1. Department of Urology, Zhongshan Hospital, Fudan University; Shanghai Key Laboratory of Organ Transplantation, Shanghai 200032, China.
2. Department of Urology, Shanghai Public Health Clinical Center, Fudan University, Shanghai 201508, China.
3. Department of Transfusion, Zhongshan Hospital, Fudan University, Shanghai 200032, China

#These authors contribute equally to the article

***Correspondence to:**

Yigang Zeng, Department of Urology, Shanghai Public Health Clinical Center, Fudan University, 2901 Caolang Road, Shanghai, 201508, China, Tel: +86-21-37990333; Email:zengyigang@shaphc.org

Tongyu Zhu, Department of Urology; Department of Transfusion, Zhongshan Hospital, Fudan University; Shanghai Key Laboratory of Organ Transplantation, 180

Fenglin Road, Shanghai, 200032, China, Tel: +86-21-64041990; Fax: +86-21-64041990; E-mail: zhu.tongyu@zs-hospital.sh.cn.

Ruiming Rong, Department of Urology; Department of Transfusion, Zhongshan Hospital, Fudan University; Shanghai Key Laboratory of Organ Transplantation, 180 Fenglin Road, Shanghai, 200032, China, Tel: +86-21-64041990; Fax: +86-21-64041990; E-mail: rong.ruiming@zs-hospital.sh.cn.

Materials and Methods

Human samples

Tumor tissue and paracancerous tissue sections were obtained from patients who were diagnosed urothelial cancer according to histology from 2016 to 2022 in Zhongshan Hospital with a history of kidney transplantation. Based on that, we performed anti-LTAg Immunohistochemistry staining assay on tumor tissue sections to identify BKV infected patients and non-infected patients. Metastasis condition was identified by PET-CT or CT scan information according to NCCN guidelines. This study was approved by the Institutional Ethics Committee of Shanghai Zhongshan Hospital, Fudan University School of Medicine (No. B2012-53).

Cells, cell culture reagents, viral stock and infection

The human umbilical vein endothelial cell (HUVEC) grew in ECM media (Cyagen, China). The human embryonic kidney cell line (HEK293) and Human bladder carcinoma cell line (HTB-9) were maintained in DMEM (Gibco, USA) in 10% fetal bovine serum. All cells were obtained from the American Type Culture Collection (ATCC; Manassas, VA, USA) and cultured in a humidified atmosphere with 5% CO_2 at 37°C.

The virus stocks initially obtained from ATCC (VR-837, Dunlop) were propagated in Vero cells with a multiplicity of infection (MOI) of 0.05. Culture medium was changed every 7 days for 3 weeks until a cytopathic effect was obvious in most cells. The scraped cells and culture medium were collected after that. We conducted

six cycles of freezing the infected cells and supernatant at − 80 °C and thawing at 37 °C to remove cell debris. Then the viral lysates were centrifuged at 10,000× g for 15 min at 4°C, and supernatant were aliquoted and store at -80°C. All BKV stocks were tittered by endpoint dilution in Vero cells.

BKPyV infections and drug treatments.

For cell counting assay, 6-well plates seeded 1×10^6 cells per well were infected with BKV at an MOI of 0, 1, 2, 4. Excess viruses were removed after 2 h of incubation. After 48 hours incubation, cells were digested by 0.25% trypsin (Gibco, USA) and centrifuged at 200g 5min. After using 1ml PBS to resuspend cells in each group, we performed cell counting using automated cell counter. The assay was repeated three times. Subsequently, HTB-9 cells seeded into 6-well plates (1×10^6 cells per well) were infected with BKV at an MOI of 1, unless indicated otherwise. Excess viruses were removed after 2 h of incubation. The cells were washed once with PBS (Corning). We included a DMSO control at a corresponding concentration in the drug treated experiments.

Plasmids, lentiviruses, and RNA oligonucleotides

The sequence of bkv-B1-miR-3p is: 5'-UGCUUGAUCCAUGUCCAGAGUC-3'; and bkv-miR-B1-5p is as following: 5'-AUCUGAGACUUGGGAAGAGCAU-3'.

The 3′UTR of CLDN1 containing bkv-miR-B1-5p binding site were cloned into the psi-CHECK-2 vector, psi-CHECK-2 empty vectors were used as control. The

predicted enhancer region containing bkv-miR-B1-3p binding locus and corresponding mutated sites were amplified using PCR from HEK293T cells and inserted into PGL3 promoter plasmid with luciferase reporter sequences for enhancer activity assays. And mutated bkv-miR-B1-3p fragment were inserted into pSUPER-GFP/NEO to construct pSR-mut-miR-3p plasmids. Renilla luciferase reporter vector pRL-SV40 was constructed for dual luciferase reporter assays. The lentivirus-bkv-miR-B1-3p and lentivirus-bkv-miR-B1-5p (HanBio, China) was purchased to generate the bkv-miR-B1-3p overexpressed or bkv-miR-B1-5p overexpressed cell lines.

Inhibitors of bkv-miR-B1-3p, bkv-miR-B1-5p and NC RNA duplex (NC) from RiboBio (Guangzhou, China) were used in this study. Three CLDN1 siRNAs (siCLDN1 #1:F:5’-AGUGGAGGAUUUACUCCUAUGTT-3’ R:5’-CAUAGGAGUAAAUCCUCCACUTT-3’, #2 F: 5’-GAUGAGGUGCAGAAGAUGAGGTT-3’; R: 5’-CCUCAUCUUCUGCACCUCAUCTT-3’ and #3,F: 5’-GAAUCGUUCAAGAAUUCUAUGTT-3’; R: 5’-CAUAGAAUUCUUGAACGAUUCTT-3’) and three importin8 siRNAs (siimportin8 #1 F: 5’-GAUAGACUAUUACUUGCAAUCTT-3’; R: GAUUGCAAGUAAUAGUCUAUCTT-3’, #2 F:5’-AGAUCAAUAUAGACAGAAAGATT-3’; R:5’-UCUUUCUGUCUAUAUUGAUCUTT-3’ and #3 F:5’-GCAUGUGAUUGGUUCCCUAGCTT-3’;

R:5'-GCUAGGGAACCAAUCACAUGCTT-3') were used separately to knock down the expression of CLDN1 and importin8 in HTB-9 and HUVECs. Plasmids, siRNAs and miRNA inhibitors were transfected using the Lipofectamine™ 3000 Transfection Reagent (Invitrogen) according to the manufacturer's instructions.

Scratch wound healing assay and transwell assays

A 12-well plate was seeded to confluence with 2×10^5 cells for wound healing assay. Scratch wounds were made using a 200 μl pipette tip. Then, medium without fetal bovine serum was used to culture the cells. Images were captured at 0h, 24h, and migration rate was calculated by ImageJ. The Transwell Permeable Supports with 8 μm pore (Corning, USA) were used to perform the cell invasion assays. The basement membrane was pretreated with 20 μg matrigel matrix (Corning, USA) and 1.5×10^5 cells/cm^2 were seeded into upper chambers. After 24 h, the transwell was fixed with 4% PFA for 15 min and stained with hematoxylin for 15 min and the cells on the upper side of basement membrane were gently removed by cotton swabs. Four random microscopic fields (magnification, 20×) were imaged for the cells on the lower side of basement membrane and the cell number was counted using ImageJ.

Dual luciferase reporter assays

After transfection of plasmids for 48 hours, the cell extracts were prepared and assayed using a Dual Luciferase Reporter Assay Kit (MCE, China) according to the manufacturer's procedures. For transfection efficiency, Renilla luciferase was used to

normalize, and the ratio of firefly/Renilla luciferase activities defined the relative activity of the enhancer region and the binding of 3'UTR or predicted enhancer region. The assay was repeated three times.

Endothelial transendothelial invasion assays

The Transwell Permeable Supports with 8μm pore were seeded separately in the top well with 1×10^6 /cm^2 HUVEC cells and in the bottom well with 1.5×10^5 /cm^2 HTB-9 cells (HTB-9 BK/HTB-9 CON), and cocultured for 72 h. Then moved the upper chamber to another well with basal medium. The top well was seeded with 1.5×10^5 /cm^2 GFP-labeled HTB-9 cells, after 24 h, the cells on the lower side of the chamber were imaged and counted.

Exosome purification, electron microscopy (EM), nanoparticle tracking analysis (NTA) and staining.

By ultracentrifugation, exosomes in cell culture medium were purified[18]. Transmission electron microscopy (TEM) and Western blotting for CD9, CD63, and TSG101 were used to identify the collected exosomes. For EM, 4% paraformaldehyde with equal volume was used to fix PBS containing exosomes, applied to the formvar-coated grids and adhered for 20 min, PBS was blotted up by filter paper gently, the grid was infiltrated by 1% uranylformate solution for 1 min and repeated two times. Images were acquired by a Transmission Electron Microscope. To estimate the particle size of exosomes, NTA was performed by NanoSight NS300 (Malvern, UK).

Exosome uptake by HUVECs

Exosomes were labelled with a green fluorescent dye (PKH67; Sigma-Aldrich, St. Louis, MO, USA) as previously described[19] and later incubated with HUVECs at 37°C for 24 hours. These cells were subsequently washed with PBS and fixed in 4% paraformaldehyde for 15 minutes. Fixed cells were washed with PBS, and nuclei were stained with DAPI (0.5 μg/mL; Invitrogen) and cytoskeleton were identified by immunofluorescence staining of β-actin. Confocal microscopy was applied to detect the signals in cells.

Animals and in vivo assays

For generating Transplanted tumor models of urothelial carcinoma in nude mice, approximately 5 × 10^6 HTB-9 cells were injected Subcutaneously in the flank of each 6-week-old male Balb/c nude mouse. After 3 weeks' feeding, the volume of initiated-tumors were measured. By using (X2Y)/2, where X = tumor width and Y = tumor length, the tumor size was calculated. To gain the tumors for further analyses, mice were sacrificed after 7 weeks. For histopathologic analyses, the standard procedure was performed. For metastasis assays, 2 × 10^6 cells resuspended in 200 μl sterile PBS were injected into the lateral tail veins of each 8-week-old male Balb/c nude mouse. After 10 weeks, mice sacrificed the livers were collected to count the metastatic nodules on surface, and then these tissues were fixed in 4% paraformaldehyde for further hematoxylin and eosin staining.

For endothelial permeability assay, 6-week-old male Balb/c nude mouse were injected with 5 × 10^5 HTB-9 cells in 250 μl sterile PBS vial tail vein. 6 hours later, 200 μl 2.5 mg/ml FITC-labeled 40-kD dextran (Sigma-Aldrich, USA) 10 min before sacrificing. For antagomir delivery, 3nmol antagomir-5p and 3nmol antagomir-3p in 200μl sterile PBS were injected into the lateral tail veins of each 4-week-old male Balb/c nude mouse 3 times per week for 4 weeks. All animal experiments were approved by the Animal Welfare & Ethics Committee of Shanghai Public Health Clinical Center Laboratory and in compliance with ethical guidelines and procedures.

Immunohistochemistry and Immunofluorescence staining analysis

On paraffin sections, immunohistochemical staining was performed. Antigen retrieval was conducted by EDTA (pH 9.0) in steam bath, then Sections were washed with TBS three times after cooling, and incubated in 3% H_2O_2 for 30 min. After that, antigen was blocked by 10% goat serum for 30 min. With primary antibodies at 4 °C overnight, the sections were labeled. Goat-anti-rabbit antibody was added to the sections on the next day. DAB was used to reveal antibody binding states. Prepared slides were photographed by ECLIPSE E600 microscope with an attached Digital Sight Camera (Nikon, Tokyo,Japan). For Immunofluorescence staining, as primary antibodies, rabbit anti-CD31 antibody (CST, 1:200), rabbit anti-SV40(CST, 1:200) were used and were separately detected by Alexa Fluor 488 donkey anti-rabbit secondary antibody (1:1000; Invitrogen) and Alexa Fluor 594 donkey anti-mouse

antibody (1:200, Invitrogen). For nuclear staining, 4′,6-diamidino-2-phenylindole (DAPI) was used.

RNA extraction, qRT-PCR

By qRT-PCR, BKPyV DNA loads were determined. The expression level of both BKV encoded large T antigen gene and VP1 gene demonstrated BKV DNA loads. Total RNA fractions were extracted with TRIzol reagent (Sigma-Aldrich). By a NanoDrop apparatus, the concentration and purity of RNA were tested. RNA with amount of 1μg was reversely transcribed into cDNA by PrimeScript RT reagent Kit with gDNA Eraser (Takara, Japan). Using Hieff qPCR SYBR Green Master Mix (high Rox) (Yeasen, China) according to the manufacturer’s instruction on An ABI QuantStudio5 platform, quantitative real-time PCR was performed. U6 was used as an internal control of microRNA related samples, and GAPDH was used as an internal control to normalize the differences in the amount of total RNA in other samples. Primers were showed in supplementary Materials.The 2−ΔΔCt method was applied as a statistical tool to calculate relative expression of each gene. The primer sequences are shown in table1.

CUT&TAG sequencing and CHIP-qPCR assay

We used 2×10^5 HUVECs incubated with extracted exosomes for 48 hours to perform this assay. Cells were immobilized on Concanavalin A-coated beads and incubated with H3K27ac antibody or IgG control in primary antibody buffer for 2 hours at room

temperature. Then beads were washed several times in wash buffer and added goat anti-rabbit Immunoglobulin G secondary antibody in antibody buffer for 1 hour at room temperature. The washing steps were repeated and added ChiTag pAG-Tn5 transposon in ChiTag buffer for 1 hour at room temperature. We activated the Tn5 transposase by adding tagmentation buffer containing MgCl2 and incubated for 1 hour at 37°C. The tagmentation reaction were stopped by adding sodium dodecyl sulfate and heating for 10 minutes at 55°C. The tagged DNA fragments were then purified using Tagment DNA Extraction Beads and amplified by PCR using Illumina-compatible primers. Using NovoNGS DNA Cleaning Beads and Qubit dsDNA HS Assay Kit, the PCR products were amplified in reactions containing 33 μl extracted DNA, 2.5 μl primer i5 (N504) and 2.5 μl primer i7 (N704), and 10 μl ×5 AmpliMix buffer. Library sequencing and data analysis were performed by OE Biotech (China).

And for CHIP-qPCR assay, after 48 hours incubation with extracted exosomes, the cells were washed twice using PBS and fixed in 1% formaldehyde for 15 min at room temperature. Then the formaldehyde was quenched by 0.125M glycine solution, and the cells were harvested and resuspended in lysis buffer (10 mM HEPES pH 7.9, 1.5 mM MgCl2, 0.5% NP-40,10 mM KCl) containing protease inhibitor cocktail (CST). Nucleus extracts were gained and dissolved in nuclear lysis buffer (50 mM Tris–HCl pH 8.1, 0.3% SDS, 10 mM EDTA and 1× cocktail). After sonication, the cell extract was incubated with antibody against H3K27ac at 4◦C for 16 h and Protein A Dynabeads (CST). Further immunoprecipitated DNA purification and qPCR assay

were conducted according to the instruction of CHIP-kit obtained from CST.

CRISPR/Cas9 system

To delete the predicted enhancer region containing bkv-miR-B1-3p locus, we purchased 6 designed gRNA for performing CRISPR/Cas9 on the certain region from HanBio, China. Through flow cytometry, transfected cells were separated to 96-well plate, cultured in 5% CO2 at 37◦C for 2 weeks, and then transferred into 24-well plate to obtain monoclonal cells. To screen the cells with efficient deletions, PCR products of the monoclonal cells were sent to Sanger sequence.

Western Blotting

In RIPA lysis buffer with protease inhibitors (Beyotime, China), cultured cells were lysed and centrifuged at 14000 rpm for 15 min at 4℃. The supernatant was separated and quantified by BCA method. On a 4–12% gradient SDS-polyacrylamide gel, protein cell lysates were loaded and then transferred onto PVDF membranes (Millipore, MA). With a primary antibody against CLDN1 (Proteintech,1/2000 dilution), SV40 (CST, 1/1000 dilution), BKPyV VP1 monoclonal antibody (Abnova, 1/500), Occludin (CST, 1/1000 dilution), TSG101 (ABClonal, 1/1000 dilution), CD9 (Abcam, 1/1000 dilution), CD63 (Abcam, 1/1000 dilution), icam-1(Proteintech,1/2000 dilution), vcam-1 (Proteintech,1/2000 dilution) , imporntin8 (CST, 1/1000 dilution) and β-Actin (CST, 1/1000 dilution) in 5% BSA,

the immunoblots were probed, followed by horseradish peroxide (HRP)-conjugated donkey anti-rabbit or anti-mouse secondary antibodies (CST,1/3000 dilution), and with an enhanced chemiluminescent system (ECL), immunostaining was detected.

Statistical Analysis

Data analysis were conducted using GraphPad Software (GraphPad Holdings, USA). Comparisons between two groups were analyzed by unpaired Student's t-test. One-way ANOVA with Tukey post hoc tests was used for comparisons between multiple groups; and two-way ANOVA was used for comparisons between multiple groups when there were 2 experimental factors. Fisher’s exact test was used to investigate the correlations between BKV LTAg staining results and distant metastasis condition or tumor pathological grade. A p-value<0.05 was considered statistically significant.

Table 1

Gene		Sequence (5'to 3')
ICAM-1	F	GGCTGGAGCTGTTTGAGAAC
	R	ACTGTGGGGTTCAACCTCTG
VCAM-1	F	TAAAATGCCTGGGAAGATGG
	R	GGTGCTCAAGTCAATGAGA
BKV-LTag	F	CTGTCCCTAAAACCCTGCAA
	R	GCCTTTCCTTCCATTCAACA
BKV-VP1	F	GGCCTCTTTGTAAAGCTGATAGC
	R	CTGTTGTGTTCCAGAGCTGTTAGTA
Bkv-B1-miR-3p	F	CGTCAGCTGTCCGAGTAGAGGTGCTTGATCCATGTC
	R	TGTCAGGCAACCGTATTCACCGACTCTG
Bkv-B1-miR-5p	F	CGTCAGCTGTCCGAGTAGAGGATCTGAGACTTGGGA
	R	TGTCAGGCAACCGTATTCACCATGCTCT
U6	F	CTCGCTTCGGCAGCACA
	R	AACGCTTCACGAATTTGCGT
CLDN1	F	GCTTCTCTCTGCCTTCTGGG
	R	TCACACGTAGTCTTTCCCGC
GAPDH	F	TGGCACCGTCAAGGCTGAGAA
	R	TGGTGAAGACGCCAGTGGACTC
CLDNCH1	F	GCTTGCCACCAAAATGTCTGGC
	R	TGTAGTAGTCATGGCAGC

Figure S1

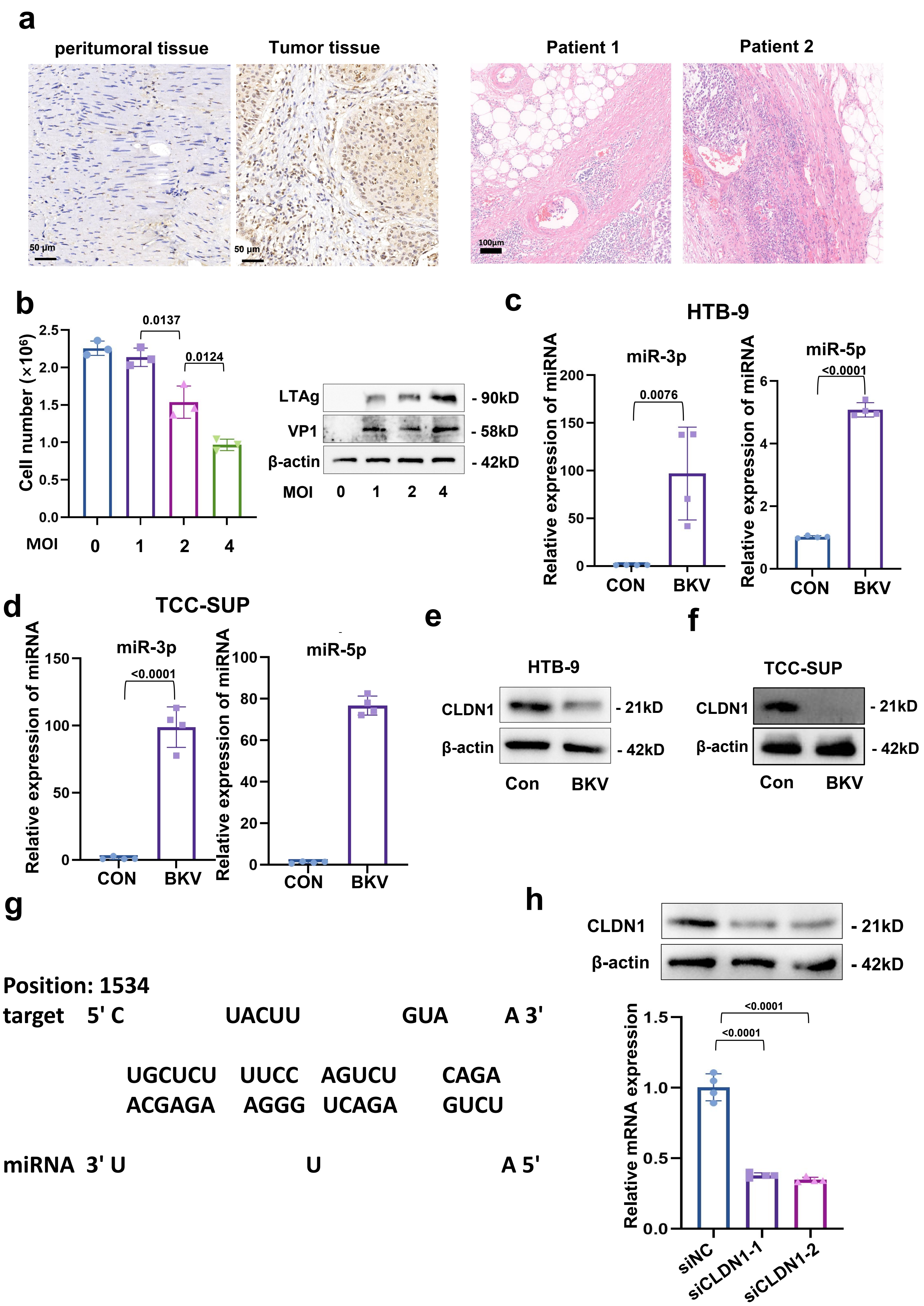

a
peritumoral tissue
Tumor tissue
Patient 1
Patient 2
50 μm
50 μm
100μm
b
Cell number (×10⁶)
2.5
2.0
1.5
1.0
0.5
0.0
0.0137
0.0124
MOI
0
1
2
4
LTAg
- 90kD
VP1
- 58kD
β-actin
- 42kD
MOI
0
1
2
4
c
HTB-9
miR-3p
miR-5p
Relative expression of miRNA
200
150
100
50
0
0.0076
<0.0001
6
4
2
0
CON
BKV
CON
BKV
d
TCC-SUP
miR-3p
miR-5p
Relative expression of miRNA
150
100
50
0
<0.0001
100
80
60
40
20
0
CON
BKV
CON
BKV
e
HTB-9
CLDN1
- 21kD
β-actin
- 42kD
Con
BKV
f
TCC-SUP
CLDN1
- 21kD
β-actin
- 42kD
Con
BKV
g
Position: 1534
target 5' C
UACUU
GUA
A 3'
UGCUCU
UUCC
AGUCU
CAGA
ACGAGA
AGGG
UCAGA
GUCU
miRNA 3' U
U
A 5'
h
CLDN1
- 21kD
β-actin
- 42kD
Relative mRNA expression
1.5
1.0
0.5
0.0
<0.0001
<0.0001
siNC
siCLDN1-1
siCLDN1-2

Figure S1 (a) IHC staining of LTAg in UCA tissues and adjacent peritumoral tissues. H&E staining shows BKV-infected tumor tissue exhibits vessel invasion and myometrial invasion. (b) WB analysis shows the LTAg and VP1 protein expressions in HTB-9 inoculating BKV at MOI of 0, 1, 2, 4. Cell survival after inoculating with BKV in different MOI. (c) RT-qPCR analysis shows the expressions of BKV-miR-3p and BKV-miR-5p are increased after HTB-9 inoculating with BKV. (d) RT-qPCR analysis shows the expressions of BKV-miR-3p and BKV-miR-5p are increased after TCC-SUP inoculating with BKV. (e) WB analysis shows the protein level of CLDN1 is decreased after HTB-9 inoculating with BKV. (f) WB analysis shows the protein level of CLDN1 is decreased after TCC-SUP inoculating with BKV. (g) The predicted binding site with highest score between BKV-miR-5p and CLDN1. (h) The mRNA level of CLDN1 in tumor cells were significantly suppressed after transfected with siCLDN1. The protein level of CLDN1 in tumor cells were significantly suppressed after transfected with siCLDN1.

Figure S2

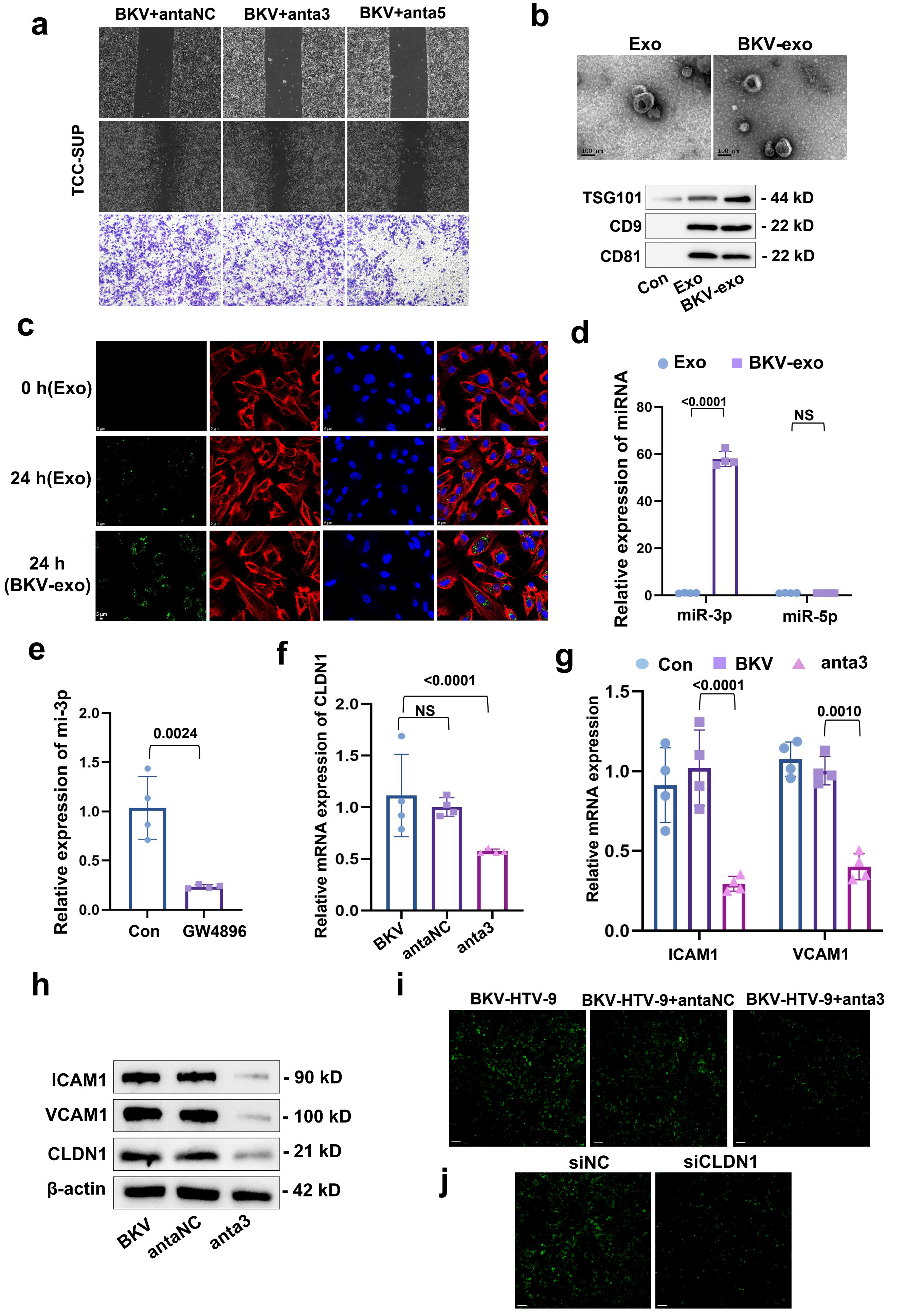
a
BKV+antaNC
BKV+anta3
BKV+anta5
TCC-SUP
b
Exo
BKV-exo
TSG101
- 44 kD
CD9
- 22 kD
CD81
- 22 kD
Con
Exo
BKV-exo
c
0 h(Exo)
24 h(Exo)
24 h (BKV-exo)
d
Exo
BKV-exo
Relative expression of miRNA
<0.0001
NS
miR-3p
miR-5p
e
Relative expression of mi-3p
0.0024
Con
GW4896
f
Relative mRNA expression of CLDN1
<0.0001
NS
BKV
antaNC
anta3
g
Con
BKV
anta3
Relative mRNA expression
<0.0001
0.0010
ICAM1
VCAM1
h
ICAM1
- 90 kD
VCAM1
- 100 kD
CLDN1
- 21 kD
β-actin
- 42 kD
BKV
antaNC
anta3
i
BKV-HTV-9
BKV-HTV-9+antaNC
BKV-HTV-9+anta3
j
siNC
siCLDN1

Figure S2 (a) Migration and invasion assays of BKV-infected TCC-SUP cells (BKV) after reducing BKV-miR-3p and BKV-miR-5p. (b) Exosomes characterization is determined by electron microscopy and WB experiment. (c) Representative images show the internalization of exosomes derived from BKV-inoculated HTB-9 (BKV-exo) and HTB-9 cells (Exo) by ECs in 0h, 24h. (d) BKV-miR-3p expression is increased in ECs after treated with BKV-exo. (e) BKV-miR-3p expression is reversed in ECs after BKV-HTB-9 pretreated with GW4869. (f) CLDN1 mRNA expression is inhibited in ECs when pre-transfected with anta3. (g) ICAM1, VCAM1 mRNA expressions are inhibited in ECs when pre-transfected with anta3. (h) CLDN1, ICAM1, VCAM1 protein expression are inhibited in ECs when BKV-HTB-9 pre-transfected with anta3. (i) HTB-9-GFP transendothelial invasion in ECs after BKV-HTB-9 pretreated with anta3. (j) HTB-9-GFP transendothelial invasion in ECs after BKV-HTB-9 pretreated with siCLDN1.

Figure S3

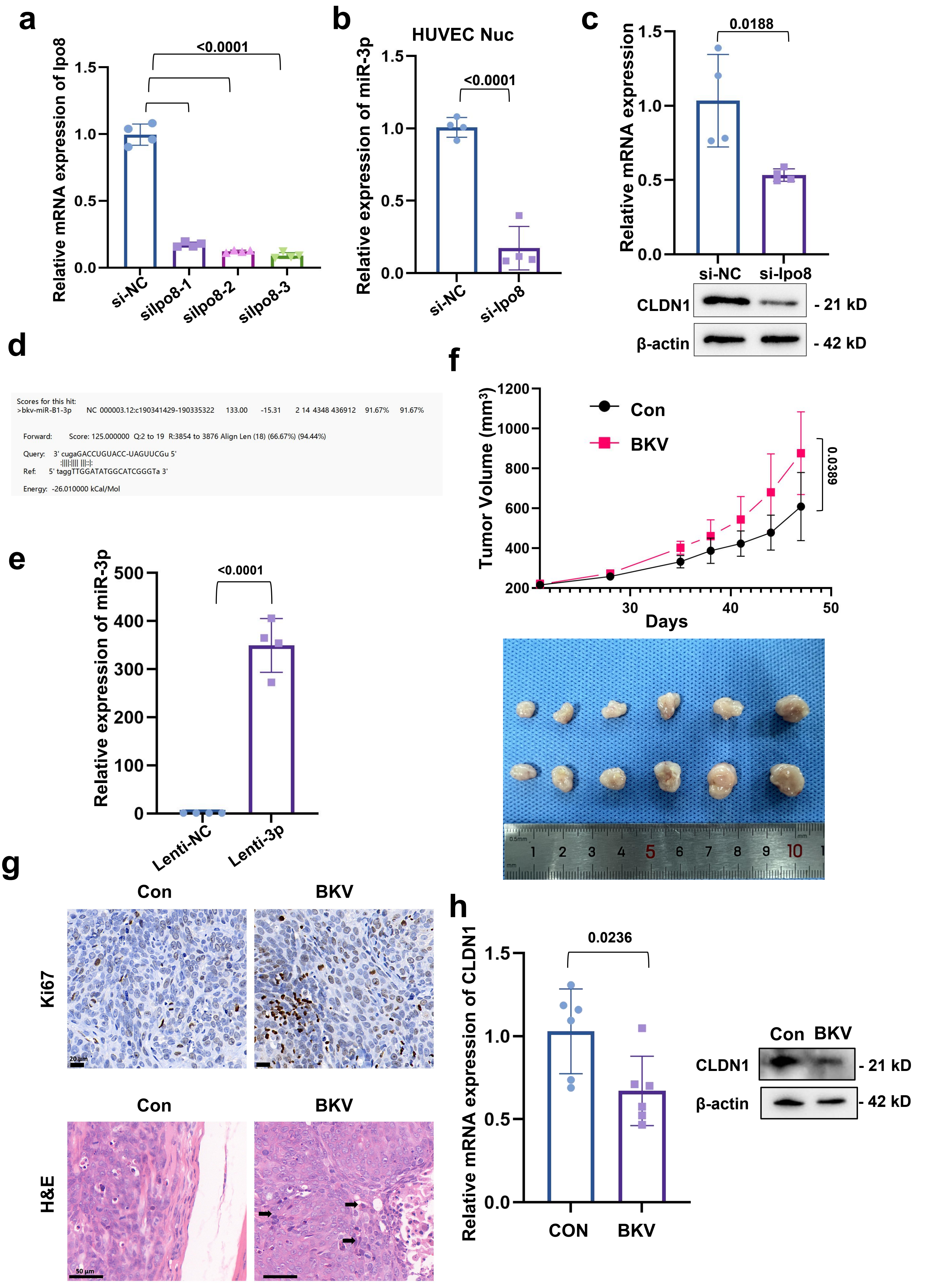
a
Relative mRNA expression of Ipo8
<0.0001
si-NC
siIpo8-1
siIpo8-2
siIpo8-3
b
HUVEC Nuc
Relative expression of miR-3p
<0.0001
si-NC
si-Ipo8
c
Relative mRNA expression
0.0188
si-NC
si-Ipo8
CLDN1
- 21 kD
β-actin
- 42 kD
d
Scores for this hit:
>bkv-miR-B1-3p NC 000003.12:c190341429-190335322 133.00 -15.31 2 14 4348 436912 91.67% 91.67%
Forward: Score: 125.000000 Q:2 to 19 R:3854 to 3876 Align Len (18) (66.67%) (94.44%)
Query: 3' cugaGACCUGUACC-UAGUUCGu 5'
Ref: 5' taggTTGGATATGGCATCGGGTa 3'
Energy: -26.010000 kCal/Mol
e
Relative expression of miR-3p
<0.0001
Lenti-NC
Lenti-3p
f
Tumor Volume (mm3)
Con
BKV
0.0389
Days
g
Con
BKV
Ki67
20 μm
H&E
50 μm
h
Relative mRNA expression of CLDN1
0.0236
CON
BKV
Con
BKV
CLDN1
- 21 kD
β-actin
- 42 kD

Figure S3 (a) The knockdown efficacy of siIpo8. (b) The expression level of bkv-miR-3p in ECs nucleus decreased after silencing Ipo8. (c) The expression level of CLDN1 in ECs is no longer promoted after silencing Ipo8. (d) The predicted results of bkv-miR-3p and CLDN1. (e) RT-PCR was employed to test the efficacy of Lenti-3p. (f) BKV promotes the tumor growth by measuring weight and volume, n=6. (g) Ki67 staining of BKV and non-BKV (Con) inoculated tumors; H&E staining for visualization of BKV and non-BKV inoculated tumor cells invading surrounding tissues in vivo, as indicated by arrows. (h) Tumors formed by BKV-inoculated cells exhibited lower expression of CLDN1 compared with non-inoculated cells, n=6.
